# How Perceived Corporate Social Responsibility Raises Employees’ Creative Behaviors Based on Appraisal Theory of Emotion: The Serial Mediation Model

**DOI:** 10.3389/fpsyg.2022.865007

**Published:** 2022-03-30

**Authors:** Said Id Bouichou, Lei Wang, Salman Zulfiqar

**Affiliations:** ^1^School of Business and Management, Donghua University Shanghai, Shanghai, China; ^2^Department of Management Sciences, COMSATS University Islamabad, Sahiwal, Pakistan

**Keywords:** perceived CSR, organizational pride, affective commitment, employee creative, appraisal theory of emotion

## Abstract

This study examines the micro-level consequences of perceived corporate social responsibility (CSR) and hypothesizes that perceived CSR affects the perception-emotion-attitude-behavior sequence. We hypothesized that perceived CSR affects organizational pride (OP) (emotion), affects affective commitment (AC) (attitude), and enhances the employees’ creative behaviors (behavior) by using the lens of appraisal theory of emotion (ATE). This study also hypothesizes that the association of perceived CSR and employee creative behaviors (ECBs) is serially mediated by OP and AC. The time-lagged data were collected from employees of only those companies participating in CSR activities to analyze the sequential mediation effect. We have tested the hypotheses of this study through Hayes approach. Results showed that perceived CSR kindles the employees’ creative behaviors. Furthermore, “organizational pride” and “affective commitment” serially mediate the association of perceived CSR and ECB. Hence, the hypothesized perception-emotion-attitude-behavior model received a significant support and demonstrated that micro-level positive consequences of CSR could be created through emotional, attitude, and behavioral mechanisms. The organization should promote their CSR activities using documentaries and contents to improve their perception of environmental and social issues, and it enhances employees’ pride and creativity.

## Introduction

Corporate social responsibility (CSR) is the process in which an organization goes beyond the financial and economic interest, corporate actions, and policies to affect the stakeholders positively (Turker, 2009a; Gond et al., 2017). With time, the concept of borderless expansion of organizational revolt results in societal concerns and organizational practices’ scandals (Wang and Bansal, 2012). These concerns outrage the research of policymakers and researchers toward CSR (Foss and Pedersen, 2016). It was observed that CSR activities have a positive impact on organizational, financial, and non-financial performance (Ali et al., 2020; Javed et al., 2020), customer loyalty (Deng and Xu, 2017), customer satisfaction (Galbreath and Shum, 2012; Saeidi et al., 2015), reputation (Park et al., 2017; Javed et al., 2020), and rate of investment.

Despite the growing number of research studies on CSR, most studies focus on the macro-level investigation. However, it is time to understand and examine CSR micro-foundations for better understanding and development of theories and to unpack the black holes present in organizational structure and practices. Besides psychological factors of individuals, this article tries to unbox the CSR practices and processes by incorporating human resources and organizational behaviors’ schemes. Bansal and Clelland (2004) empirically analyzed and examined the CSR activities to understand their micro-foundations. Aguinis and Glavas (2012) empirically investigated the impact of CSR on employees’ work engagement and enthusiasm. In contrast, Gond et al. (2017) examined the impact of CSR on the emotional and cognitive appraisal of the people. Glavas and Kelley (2014) and Barakat et al. (2016) examined employees’ organizational CSR perception and job satisfaction. Gao and He (2017) measured organizational citizenship behavior, and Kim et al. (2010) extracted organizational commitment in this regard. DeRoeck et al. (2016) examined the CSR perception relationship with organizational identification through serial mediation of perceived external prestige and organizational pride (OP).

The CSR micro-foundation is important to comprehend and understand the organization’s employee perception regarding CSR activities. Perceived CSR is defined as the viewpoint of employees regarding the CSR activities in which an organization participates (Akremi et al., 2018). It also covers the employee perception regarding CSR activities rather than the firm’s objective perspective. Both objective and perceived CSR are determinants of perceived CSR, but perceived CSR is a more appropriate dimension to understand and examine the micro-foundation of CSR (Rupp and Shao, 2013). According to the studies by Morgeson et al. (2013) and Newman and Brucks (2018), to get meaningful intrapsychic reactions of employees, organizations need to take measurable steps to be aware of CSR practices carried out by the organization. Perceived CSR shapes emotion, attitude, and behavior and builds a positive image of the organization in employees’ eyes. Employees are the organization’s stakeholders who act upon, perceive, evaluate, and react upon the CSR activities performed by their organization (Akremi et al., 2018). Valentine and Godkin (2016) described the perceived CSR as predictors of work outcomes, such as productivity, performance, job satisfaction, and turnover intention.

Based on the literature, it was investigated that the researcher uses only a few conceptual frameworks to explain the association between the perceived CSR and attitude and behavior of the employee. Glavas and Kelley (2014) identified that the gap between CSR and employee outcome encouraged researchers and policymakers to discover a possible association mechanism. However, Ng et al. (2019) presented a theoretical framework using affect-based theories to comprehend the relationship between perceived CSR and employee outcomes. To fill the present gap in this study, the theoretical framework of the appraisal theory of emotions (ATE) is adopted, which will explain the relationship between perceived CSR and employee creativity. To enhance the understanding of CSR micro-foundation, this study hypothesizes the conceptual framework of perception-emotion-attitude-behavior in sequence. This means that perceived CSR leads to employee emotion, i.e., OP. These emotions trigger the attitude of the employees, i.e., organizational commitment. Finally, attitude results in behavior, i.e., employee creative behaviors (ECBs).

In this study, we tried to build a linkage between the perceived CSR and the emotional part of an individual, namely, OP, attitude part of the organizational commitment, and behavioral part of the ECB. Weiss and Cropanzano (1996) presented why individuals’ outcomes are affected due to perceived CSR. Various types of emotion are considered for enhanced outcomes. This study focuses on OP because it measures the tendency of individuals to get sensible cues that make them feel good about themselves or the group they belong to Roccas et al. (2008). Pfeffer and Fong (2005) examined that an individual with hedonic wellbeing expects positive information and looks forward to organizational cues.

This article chooses organizational commitment as a job attitude to construct because it covers the psychological linkage of an individual with their organization and continues to have a positive relationship with the organization (Farrukh et al., 2017). Organizational commitment is how employees show their support and loyalty toward the organization. According to the study by Joiner and Bakalis (2006), organizational commitment is an important aspect of attitude because it effectively fosters the working environment of the organization.

At the final stage, this article focuses on ECB as a dependent variable because it impacts organizational initiatives and outcomes. Employees with high creativity enhance organizational performance (Raza et al., 2021). Tierney et al. (1999) defined creativity as unique and useful solutions generated by employees while responding to their work-related problems based on the visions and mission of the organizations. The creative behavior of employees provides opportunities to come up with new and innovative solutions to the problems faced by the organization (Ahmed, 1998; Martins and Terblanch, 2003; Gumusluoglu and Ilsev, 2009; Pitta, 2009; Moghimi and Subramaniam, 2013). If perceived CSR enhances employees’ creative behavior, then the importance of CSR readily becomes apparent. This means that organizations need to adjust and redesign their social initiatives valuable for their stakeholders, especially for the employees.

The CSR activities are an important aspect for promoting sustainable firm performance; it is time to understand and comprehend CSR micro-foundations. It is important to recognize and apprehend the effects of perceived CSR on ECB. In this study, we examined the perception-emotion-attitude-behavior model to unravel the intrapsychic association between the variables that are perceived CSR and employee outcome.

### Importance of Corporate Social Responsibility

Regardless of CSR significance, exploration, and investigation, researchers are still unable to reach the commonly accepted definition of CSR. Researchers describe CSR from different perspectives, such as social performance (Barakat et al., 2016), corporate governance (Shalley, 1995), stakeholder management (Stites and Michael, 2011), and corporate citizenship (Gao and He, 2017). The widely and commonly cited definition of CSR is the economic, ethical, and discretionary expectation of the society from the organization at the given time (Carroll, 1979). Matten and Moon (2008) explained that CSR is the organization’s economic, legal, and technical obligation toward society. Rupp et al. (2006) explained CSR as the organization’s policies, actions, and decisions for the wellbeing and sustainability of the society. Turker (2009b) classified CSR into four main categories, which include CSR activities toward (1) social and non-social stakeholders, (2) employees, (3) customers, and (4) the government.

The CSR activities toward social and non-social stakeholders are that organizations indulge in activities to serve society and the environment and to make the generation better. CSR toward employees is that organizations ensure and engage in activities for the wellbeing of the employees, including job security, equal opportunities for growth, work-family balance, and flexible environment. At the same time, CSR for the customer is the responsibility of businesses toward customer satisfaction, such as providing them quality products, after-sale services, and customer care.

### Theoretical Support and Hypothesis Development

Eagly and Chaiken (1998) categorized emotions and attitudes as different psychological constructs but, at the same time, highlighted the relationship between emotions and attitudes by definition. Elfenbein’s (2007) emotions are the source for attitude and behavior; in other words, attitudes are post-emotional responses. To better understand the relationship between attitudes and emotions, one needs to comprehend the dimensions of attitude. Two main domains of attitudes are affective and cognitive domains. Berg et al. (2006) described that affective domain is defined as the emotions and feelings of individuals toward a certain object, whereas the cognitive domain is defined as an individual’s beliefs and thoughts toward a certain object. The ATE strives to comprehend the factors that define the individual’s emotional experience from the cognitive perspective (Glavas and Kelley, 2014; Brosi et al., 2018). The key precept of the theory is the appraisal of the event, rather than objective reality, which determines the individual’s emotional experience. In other words, similar stimuli determine different emotional responses among different individuals.

To study the emotions and attitudes of individuals, a recommended framework is ATE which was developed by Weiss and Cropanzano (1996). Using ATE, Basch and Fisher (2000) explained that workplace structure, such as leadership style and job design, influences the occurrence of stimulus, which directly impacts the work actions and proceedings. Experiencing these actions and events leads to positive or negative work emotions. Both workplace structure and emotions impact the employee’s emotions affiliated with the organization. Typically, this model considered workplace attitude, which results in job satisfaction (Smith et al., 1992).

It is suggested that affect-driven behaviors, such as positive or negative citizenship behaviors and employee problem-solving behaviors, are influenced through emotions (Gouthier and Rhein, 2011; Kraemer and Gouthier, 2014; Brosi et al., 2018). ATE merges the domains of emotions and attitude to study the human behavior at work. Arena et al. (2018) mediated the role of emotions to stabilize the features of the working environment, which impacts the attitude and behavior of an individual at work. The primary focus of this study is to investigate the relationship of emotions and attitude that is the impact of perceived CSR on OP which affects the organizational commitment and leads to creative behavior.

**FIGURE 1:**
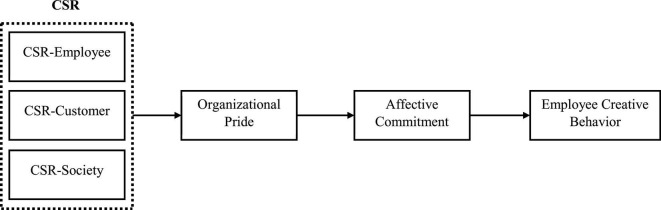
Theoretical framework.

In this study, we suggested CSR’s cognitive and affective emotional appraisal, which explains the employee’s emotional response to CSR activities. Firms’ CSR activities are often described in financial reports, official websites, social media official channels, and statements by shareholders. According to the study by Lazarus and Folkman (1987), individuals must be aware of the stimuli that appraise their emotional response for cognitive appraisal. Most employees in the organizations are unaware of the CSR activities performed by their organization. Moreover, employees perceive CSR activities differently and appraise them differently. For instance, some employees in the organization consider the CSR activities performed by the organization as a stunt to build the relationships rather than for the wellbeing of society. The appraiser reactions are influenced by the individual subjective perception of CSR, such as purpose, motivation, and beneficiaries of CSR activities (Glavas and Kelley, 2014; Farooq and Salam, 2020). Furthermore, individual perception affects the situation’s feelings, thoughts, and reactions (Dögl and Holtbrügge, 2014; Roeck et al., 2014). It is suitable to use a cognitive appraisal perspective to investigate this study based on the literature (Figure 1).

At present, researchers focus on the macro-level perspective of CSR; there is a significant gap in studying the micro-perspective of CSR. It is depicted that with cognitive and affective appraisal, individual emotional linkages with the organization change, leading to change in attitude and behavior of the employee. According to the study by Ballinger and Schoorman (2007), emotions lead to job attitude, i.e., perceived CSR leads OP, and then this positive attitude cultivates positive work behavior, which motivates employees’ creative behavior.

### Perceived Corporate Social Responsibility and Organizational Pride

The OP is defined as the degree to which an employee experiences a sense of pleasure and his/her self-esteem aroused due to organizational affiliation. Pride emerges when employees are given information and cues regarding organizational CSR activities that assist them in appraising their membership with their organization. Shariff and Tracy (2014) described that employees get motivated and their sense of pride enhanced, which results in self-esteem and social pride. Stites and Michael (2011) examined that pride arouses cognitive appraisal, and it is important to understand the individual reaction toward CSR activities. Ng et al. (2019) explained that employee pride enhances when they get the sensation that their organization is doing for the wellbeing of society beyond what the average firm can do. At the same time, perceived CSR is a surety given by the organization that they will dedicate resources and efforts to serve the interest of the stakeholders. Pride stimulates when the employee feels that they are being associated with a responsible and competent organization that assists society in improving and progressing. At present, due to globalization, employees have ideological values and desire to have organizational CSR activities that impact society. Based on the above literature, we proposed the following hypothesis:

H1: Perceived CSR has a significant effect on employees’ OP.

### Organizational Pride and Organizational Commitment

John et al. (2019) explained that positive emotions, such as OP, inflate thoughts, and actions contribute to open-mindedness among the employees, enhancing their thirst for new information and knowledge (Farooq and Salam, 2020). Therefore, proud employees exhibit a progressive commitment toward the organization and exert their efforts and energies for the progression and development of the organization (John et al., 2019). Employees who commit their efforts toward organization goals look for high quality and creative ideas. Organizational commitment is the employee’s psychological connection with the organization (Guterman, 1974; Jones, 2010). Organizational commitment assists in developing a variety of positive antecedents of organizational behavior; in other words, Ng et al. (2019) explained that the higher the organizational commitment, the higher the work creativity and performance and the lower the turnover ratio. In post-industrial and knowledge-based societies, organizations focus on creativity for the organization’s long-term development, so the employees who have strong emotional and psychological attachment with the organization perform innovatively and creatively to achieve organizational goals. Minor and Morgan (2011) explored the relationship between organizational commitment and workplace creativity using qualitative and quantitative methods. During the study period, they interviewed a supervisor from a technology-based organization; interviewees exemplify that committed employees are always looking for innovative and creative ways to improve business operations and achieve organizational goals. Tsachouridi and Nikandrou (2016) urged a positive relationship between organizational commitment and workplace creativity. Based on the earlier arguments, we proposed the following hypothesis:

H2: Employees’ OP has a significant association with employees’ affective commitment (AC).

### Perceived Corporate Social Responsibility and Workplace Creativity

According to the study by Franken (1994), creativity is defined as a tendency to produce useful ideas, thoughts, products, and services, looking for possible alternatives to solve the problem. Due to technological advancements, workstation environment changes and organizations emphasize workplace creativity (Moghimi and Subramaniam, 2013). Workplace creativity works as the engine of change, which provides opportunities for organizations to flourish and grow (Ahmed, 1998; Martins and Terblanch, 2003; Gumusluoglu and Ilsev, 2009; Pitta, 2009; Moghimi and Subramaniam, 2013). Tierney et al. (1999) defined creativity as unique and useful solutions generated by employees while responding to their work-related problems based on the visions and goals of their organizations. George and Zhou (2001) described that workplace creativity is the production of valuable and innovative ideas that assist organizations in improving and progressing toward their defined goals. A creative workplace is instrumental for employees themselves and their organizations. Franken (1994) clarified that the employee’s commitment to the organization results in workplace creativity, which motivates them to find novel and contemporary solutions to the problems (Mishra and Shukla, 2012).

The attitude and behavioral response of the employees are greatly influenced by their perception of organizational action rather than actual behavior (Rupp et al., 2006). According to Akremi et al. (2018), CSR is the organization’s actions and policies to boost the prosperity and wellbeing of the stakeholder by accounting for economic, environmental, and social performance. Tajfel and Turner (1985) examined that organizations involved in CSR activities are considered worthy corporate citizens, and employees feel more ethically strong with such organizations. Employees in such an organization feel the organization’s success and show their creative behavior to achieve organizational goals. Attitude and behavior of such employees result in high persistence, risk taking, openness to experience, creative, and great efforts (Amabile et al., 2005; Grant and Berry, 2011). Employees working in socially responsible environments think out of the box and develop innovative and creative ideas to solve social problems (Brammer et al., 2015). An organization that indulges in CSR activities provides an unrestricted and safe environment that encourages employees to take risks and perform experiments to produce noble and novel goods and services to the stakeholders (Zhou and George, 2001). Organizations are concerned about the wellbeing of society and intrinsically motivate employees to solve social problems creatively (Hur et al., 2018). Intrinsic motivation predicts an employee’s involvement in creative and innovative activities (Chaudhary and Akhouri, 2019). Based on the above literature, we proposed the following hypotheses:

H3: Perceived CSR has a significant impact on ECB.

H4: OP and AC serially mediate the association of perceived CSR and employee’s creative behavior.

## Methodology

### Sample and Procedure

In this study, data were collected from employees working in companies listed on the Morocco stock exchange. We included employees of different industries in our sample size to better understand the role of CSR in employees’ creative behavior. Furthermore, time-lagged data were collected from employees of only those companies who were participating in CSR activities. We also confirmed the companies’ involvement in CSR activities from their websites and annual reports. The higher authority of each company was contacted using personal references to seek permission for data collection. The human resource department was asked to provide the employees’ list for survey distribution. The data were collected by using a structured questionnaire. Each questionnaire was started with a cover letter, which includes the consent section, explanation about research goal, and confirmation of participants’ confidentiality. The pre-remedial strategies recommended by Spector and Brannick (1995) were adopted to address the potential issue of common method bias. Similarly, the participants were asked to answer honestly, to follow the guidelines to complete the survey, and to read the instructions that questions have no right or wrong answer. After the cover letter, the next section contained questions about employees’ CSR perception, OP, organizational commitment, and employees’ creativity. The last section consisted of demographic information (e.g., participants’ gender, education, age, and experience in the current organization). We developed the questionnaire by using each variable’s cited, reliable, and valid measurement scales. Laterally, the first draft of the questionnaire was shared with 10 academicians to ensure scales’ face and content validity. The academicians suggested some changes to improve the clarity of participants’ questionnaire and helped finalize the survey. In the first stage, 500 questionnaires related to perceived CSR were randomly distributed among the employees, and 410 respondents returned with the questionnaire with a response rate of 82%. Each participant was assigned a unique number to contact them at the second and third stages. However, each participant was assured that identity would not be shared with anyone, and responses will be reported in aggregate form. After 4 weeks, we contacted employees who responded at the first stage and asked them to answer the questions related to OP and organizational commitment. A total of 320 respondents answered the questions at the second stage with a response rate of 78%. After 4 weeks of interval, participants of the second stage were again contacted and asked to respond to creative behavior and demographics. A total of 294 respondents returned the questionnaire at the third stage, and 6 were deleted due to incomplete information. Finally, 288 questionnaires were used for final analysis with a response rate of 58%.

The demographic results showed that 167 (58%) participants were men, and more than 60% (177) employees worked in the current organization for 5 and more years. This shows that participants were well aware of organizations’ CSR activities and better positioned to respond to the questions. The results also reported that the average age of the participants was 27.61 years. The detailed results of respondents’ demographics are presented in Table 1. The recommended procedure (Brislin, 1970) was used to translate and translate back from English to Arabic for the survey participants.

**TABLE 1:** Demographic analysis.

Demographic variable	Categories	Frequency
Gender	Female	121 (42%)
	Male	167 (58%)
Education	Under graduation	46 (16%
	Graduation	132 (46%)
	Master/M.Phil	75 (26%)
	Professional certification	35 (12%)
Age	21–25 years	32 (11%)
	26–30	104 (36%)
	31–35	118 (41%)
	36–40	23 (8%)
	Above 40 years	11 (4%)
Experience in current organization	1–4 years	95 (33%)
	5–8	135 (47%)
	9–12	38 (13%)
	Above 12 years	20 (7%)

### Measurements

The constructs of this study were adapted from literature and measured on the 5-point Likert-type scale (“1 = strongly disagree” to “5 = strongly agree”). We chose the 5-point Likert-type scale as it reduces the participant’s annoyance and enhances data quality (Nauman et al., 2019; Ali et al., 2020). Numerous steps were taken for the selection of measurement items. At the first stage, well-established constructs were translated from English to Arabic and shared with academicians for required modification. All measurement scales were finalized after the modification suggested by the academicians at the pretested stage to confirm that scales are the best fit to study context. The recommendations of Brislin (1970) are followed for back translation to ensure that translation bias did not exist in our Arabic questionnaire version. We also confirmed the construct’s validity and reliability through composite reliability (CR) and confirmatory factor analysis.

### Perceived Corporate Social Responsibility

The fourteen-item scale of Turker (2009b) was adapted to measure the perceived CSR toward key stakeholders (e.g., society, employee, and customer). The participants were asked to disclose how much they agreed with the statement on the 5-point Likert-type scale. Example items include “our company emphasizes the importance of its social responsibilities to the society,” “our company policies encourage the employees to develop their skills and careers,” and “our company provides full and accurate information about its products to its customers.” The previous researchers have used this scale in their studies (Wang et al., 2020). The scale’s reliability was confirmed through Cronbach’s alpha (α = 0.86), and the statistics were above the threshold value (Cortina, 1993). The factor loading in the range of 0.72–0.85 established convergent validity, and discriminant validity was confirmed through average variance extracted (AVE), which was more than 0.50 (Hair et al., 2010). The model fitness indices proved that all items yield good fit model [comparative fit index (CFI) = 0.91, root mean square root error of approximation (RMSEA) = 0.47, standardized root mean squared residual (SRMR) = 0.054, CMIN/*df* = 3.10].

### Organizational Pride

The OP has been measured using a four-item scale developed by Cable and Turban (2003). Example items include “I am proud to be part of this organization” and “I feel proud to identify myself personally with this organization.” Helm (2013) also used this scale in her study. We have confirmed reliability through Cronbach’s alpha (α = 0.81). Convergent validity proved through factor loading in the range of 0.68–0.75, and discriminant validity confirmed through AVE (Hair et al., 2010). The four items fitted the best model fit as indicated by fitness indices (CFI = 0.93, RMSEA = 0.051, SRMR = 0.047, CMIN/*df* = 2.96).

### Affective Commitment

The six-item scale has been used to measure the AC of employees (Allen and Meyer, 1990). The previous studies (Ng et al., 2019; George et al., 2020) have used the same scales to measure AC. The sample questions include “I feel emotionally attached to this organization” and “I would be very happy to spend the rest of my career with this organization.” The scale’s reliability was established through Cronbach’s alpha (α = 0.78), and the statistics were above the threshold value (Cortina, 1993). The factor loading ranging from 0.70 to 0.82 confirmed convergent validity, and discriminant validity was established through AVE, more than 0.50 (Hair et al., 2010). The six items fitted the best model fit as indicated by fitness indices (CFI = 0.95, RMSEA = 0.041, SRMR = 0.063, CMIN/*df* = 2.81).

### Employees’ Creative Behavior

A thirteen-item scale recommended by Zhou and George (2001) has been used to measure ECBs. The supervisors of selected companies were asked to rate their employees’ creative behaviors on a 5-point Likert-type scale. The sample items include “our employees suggest new ways to achieve goals or objectives” and “our employees promote and champion ideas to others.” This scale is well established and used in many studies (Abdelmotaleb et al., 2018; Duarte et al., 2021). We have confirmed reliability through Cronbach’s alpha (α = 0.86). Convergent validity proved through factor loading in the range of 0.68–0.86, and discriminant validity confirmed through AVE (Hair et al., 2010). The thirteen items fitted the best model fit as indicated by fitness indices (CFI = 0.96, RMSEA = 0.047, SRMR = 0.056, CMIN/*df* = 2.82).

### Control Variables

The previous researchers (Sarker et al., 2011; Abdelmotaleb et al., 2018) have identified employee tenure and age as controlled variables because these variables are related to employees’ behaviors. We also controlled the effect of these variables in this study. However, the statistical results indicated that these controlled variables are not related to this study’s dependent variable. Therefore, we dropped control variables from further analysis by following the guidelines.

### Confirmatory Factor Analysis

The CFA was conducted to evaluate the reliability and validity of the constructs of this study. It is recommended and endorsed to assess construct validity (Hair et al., 2010; Byrne, 2016). The convergent validity was established through factor loading, which should be above 0.50 as recommended by many researchers (Fornell and Larcker, 1981; Hair et al., 2010). The factor loading of items was above the threshold value (Table 2) that proved the convergent validity of constructs. However, a few CSR and ECBs items were deleted because they were not meeting the threshold limit of factor loadings (> 0.50). The convergent validity was also assessed through AVE. The results show that AVE is above the cutoff value, as presented in Table 2. Therefore, the convergent validity of all constructs is approved.

**TABLE 2:** Validity and reliability.

Items	Estimate	AVE	CR
**CSR-employee**			
CSR_Emp1	0.76		
CSR_Emp2	0.79		
CSR_Emp3	0.80		
CSR_Emp4	0.72		
CSR_Emp5	0.74	0.58	0.87
**CSR-customer**			
CSR_Cus1	0.78		
CSR_Cus2	0.74		
CSR_Cus3	0.84	0.62	0.83
**CSR-society**			
CSR_Soc1	0.75		
CSR_Soc2	0.81		
CSR_Soc3	0.77		
CSR_Soc4	0.84		
CSR_Soc5	0.80		
CSR_Soc6	0.73	0.615	0.90
**Organizational pride**			
OP1	0.71		
OP2	0.75		
OP3	0.72		
OP4	0.68	0.51	0.80
**Affective commitment**			
AC1	0.70		
AC2	0.76		
AC3	0.82		
AC4	0.74		
AC5	0.78		
AC6	0.71	0.56	0.88
**Employee creative behavior**			
ECB1	0.75		
ECB2	0.72		
ECB3	0.70		
ECB4	0.83		
ECB5	0.74		
ECB6	0.73		
ECB7	0.67		
ECB8	0.71		
ECB9	0.72		
ECB10	0.69		
ECB11	0.73		
ECB12	0.70		
ECB13	0.67	0.519	0.93

*AVE, Average Variance Extracted; CR, Composite Reliability; CSR_Emp, CSR toward employee; CSR_Cust, CSR toward customer; CSR_Soc, CSR toward society; OP, Organizational Pride; AC, Affective Commitment; ECB, Employee Creative Behavior.*

The square root of AVE was used to assess the discriminant validity (Fornell and Larcker, 1981), and it should be above the inter-construct correlation. The results showed (Table 3) that the square root of AVE was above the correlation of variables. Thus, the discriminant validity was also confirmed (Hair et al., 2010). The constructs’ reliability was confirmed through CR and Cronbach’s alpha (α). The values of CR and α were also above the cutoff point (Table 2), which approved the reliability of the construct.

**TABLE 3:** Correlational analysis.

	Mean	SD	*A*	CSR_Emp	CSR_Cust	CSR_Soc	OP	AC	ECB
CSR_Emp	3.97	0.67	0.86	***(0.76)***					
CSR_Cust	3.84	0.74	0.80	0.67	***(0.79)***				
CSR_Soc	3.92	0.71	0.78	0.61	0.66	***(0.78)***			
OP	3.78	0.83	0.81	0.56	0.59	0.64	***(0.71)***		
AC	3.91	0.75	0.78	0.54	0.50	0.56	0.63	***(0.74)***	
ECB	3.87	0.80	0.86	0.36	0.47	0.44	0.52	0.49	***(0.72)***

*CSR_Emp, CSR toward employee; CSR_Cust, CSR toward customer; CSR_Soc, CSR toward society; OP, Organizational Pride; AC, Affective Commitment; ECB, Employee Creative Behavior.*

*The value in () on diagonal represents the square root of AVE.*

The model fitness indices reported (Table 4) that the fitness of six factors’ full model is the best fit (CMIN = 1559.06, *df* = 545, CMIN/*df* = 2.86, RMSEA = 0.075, CFI = 0.931, GFI = 0.921, and Tucker-Lewis index (TLI) = 0.915) as compared to one factor model which is poor fit (CMIN = 8687.39, *df* = 615, CMIN/*df* = 14.12, RMSEA = 0.258, CFI = 0.38, GFI = 0.35, and TLI = 0.27).

**TABLE 4:** Model fitness of measurement model.

	Measurement Models (CFA)	CMIN	df	CMIN/df	RMSEA	CFI	GFI	TLI
1	One factor model of corporate social responsibility	1082.15	77	17.43	0.231	0.578	0.554	0.501
	Three factor model of corporate social responsibility	**360.62**	**74**	**4.87**	**0.102**	**0.829**	**0.80**	**0.789**
2	One factor model of both mediators (organizational pride and affective commitment)	398.45	35	11.384	0.206	0.746	0.748	0.673
	Two factor model of both mediators (organizational pride and affective commitment)	**85.159**	**26**	**3.314**	**0.097**	**0.953**	**0.92**	**0.935**
3	One factor model of organizational pride, affective commitment and employee creative behaviors	1368.34	209	6.547	0.151	0.663	0.563	0.628
	Three factor model of organizational pride, affective commitment and employee creative behaviors	**689.49**	**186**	**3.707**	**0.105**	**0.846**	**0.764**	**0.827**
4	Complete model (one factor)	8687.39	615	14.12	0.258	0.38	0.35	0.27
	Complete Model (six factor)	**1559.06**	**545**	**2.86**	**0.075**	**0.931**	**0.921**	**0.915**

*Bold indicates alternate model testing.*

### Common Method Bias

The authors have used different methods to curtail the potential issues of common method bias. First, we have adopted pre-remedial strategies at the time of data collection recommended by Spector and Brannick (1995). Second, the authors used the single Harman test to confirm whether common method bias exists or not. The result suggests that common method bias may not be an issue as a single factor explains only 38.09% variance less than the threshold value of 50%. Third, we conducted CFA of all variables included in the hypothesized model. The CFA results showed (Table 4) that model fitness indices are above the threshold value recommended by Hair et al. (2010) of the nine-factor full model compared to the one-factor model whose fitness is compared to the one-factor model indices below the recommended value. All these methods suggest that common method bias may not be the issue of this study.

### Descriptive Analysis

Before testing the hypotheses, descriptive and correlational analysis was performed to ascertain the central tendency, deviations, and bivariate relationship of studied constructs. Results showed that CSR dimensions (“CSR toward employees, customer and society”) have a positive significant correlation with OP (*r* = 0.56, 0.59, 0.64), AC (*r* = 0.54, 0.50, 0.56), and ECB (*r* = 0.36, 0.47, 0.44). OP is significantly correlated with AC and ECB (*r* = 0.63, 0.52). Furthermore, AC is positively correlated with ECB (*r* = 0.49). The Cronbach’s α was used to assess the variables’ reliability (internal consistency). The threshold value of α is 0.70 as recommended by Clark and Watson (1995). The statistics depict that the α value of all variables is above the threshold value. Results of the descriptive and correlational analysis are shown in Table 3.

### Hypothesis Testing

We tested the hypotheses of direct and indirect effect and serial mediation by using the SPSS macro (PROCESS) developed by Hayes (2013). PROCESS model 4 is used for simple mediation, and model 6 is applied for serial mediation (Table 5). The results indicated that CSR’s dimensions have a positive significant direct effect on OP (β = 0.64, *p* < 0.001; β = 0.68, *p* < 0.001; β = 0.52, *p* < 0.001), AC (β = 0.72, *p* < 0.001; β = 0.54, *p* < 0.01; β = 0.63, *p* < 0.001), and ECB (β = 0.45, *p* < 0.05; β = 0.34, *p* < 0.05; β = 0.29, *p* < 0.05). OP and AC also have a positive significant impact on ECB (β = 0.66, *p* < 0.001; β = 0.59, *p* < 0.001).

**TABLE 5:** Hypothesis analysis.

	Organizational pride			Affective commitment			Employee creative behavior		
	β	SE	*R* ^2^	B	SE	*R* ^2^	β	SE	*R* ^2^
CSR-employee	0.64***	0.217	0.33	0.72***	0.258	0.30	0.45*	0.211	0.37
CSR-customer	0.68***	0.099	0.521	0.54**	0.090	0.40	0.34*	0.077	0.56
CSR-society	0.52***	0.118	0.304	0.63***	0.085	0.551	0.29*	0.070	0.63
Organizational pride				0.68***	0.224	0.33	0.66***	0.198	0.39
Affective commitment							0.59***	0.166	0.41
**Indirect effects (perceived CSR-ECB)**						Effect [95% LLCI, ULCI] and Sobel (*z*-value)			
CSR_EmpOPECB							*0.26 [0.1323, 0.4990], (3.56)*		
CSR_CustOPECB							*0.28 [0.1571, 0.4631], (3.55)*		
CSR_SocOPECB							*0.21 [0.1332, 0.3484], (3.60)*		
CSR_EmpACECB							*0.29 [0.1330, 0.5034], (3.41)*		
CSR_CustACECB							*0.24 [0.1145, 0.4171], (3.44)*		
CSR_SocACECB							*0.20 [0.0885, 0.3843], (2.82)*		
**Serial mediation**									
CSR_EmpOPACECB							0.215 [0.0920, 0.4924]		
CSR_CustOPACECB							0.108 [0.208, 0.2697]		
CSR_SocOPACECB							0.209 [0.0979, 0.3811]		

*CSR, Corporate Social Responsibility; OP, Organizational Pride; AC, Affective Commitment; ECB, Employee Creative Behavior.*

*Results are reported at 95% CI by using 5,000 bootstrapping sample.*

***Significant at 0.001, **Significant at 0.01, *Significant at 0.05. Direct and indirect mediation are indicated in italics.

The results (Table 5) of mediation show that OP mediates the relationship of CSR dimensions and ECB [β = 0.26, CI (0.1323, 0.4990), *z* = 3.56; β = 0.28, CI (0.1571, 0.4631), *z* = 3.55; β = 0.21, CI (0.132, 0.3484), *z* = 3.60]. Furthermore, AC mediates the association of CSR dimensions and ECB [β = 0.29, CI (0.1330, 0.5034), *z* = 3.41; β = 0.24, CI (0.1145, 0.4171), *z* = 3.44; β = 0.20, CI (0.0885, 0.3843), *z* = 2.82]. Furthermore, OP and AC serially mediate the CSR dimensions and ECB association, respectively. The statistics of PROCESS model 6 reported a significant indirect effect [β = 0.215, CI (0.0920, 0.4924); β = 0.108, CI (0.0208, 0.2697); β = 0.209, CI (0.0979, 0.3811)]. Therefore, the serial mediation hypotheses are supported.

## Discussion

This study aims to verify the proposed model, which comprehends how perceived CSR affects the employees’ pride and commitment, leading to creative behavior among the employees, i.e., incorporating the ATE. In this study, the authors investigated the direct relationship between perceived CSR and creative work behavior and serial mediation of OP and organizational commitment. This study suggests that implementing CSR activities enhances OP among the employees. Furthermore, this study extends the literature and assists researchers to enhance pro-environmental behavior because of the CSR activities adopted by the organization.

To understand the mechanism of perceived CSR and creative work behavior, this study empirically identifies the potential mediators. We examined that perceived CSR and creative behavior are not simple to understand, and it follows multiple sequential mediators, i.e., OP and organizational commitment. CSR becomes an important corporate sustainable policy for organizations, and this study comprehends the mechanism that how and when perceived CSR activities influence employee behavior particularly affecting the creative working environment.

This research considers OP and organizational commitment to fill in the gap. When organizations indulge in CSR activities and focus on environmental concerns, these activities enhance the image and reputation of the organization in the eyes of the stakeholder, particularly employees, which results in employee pride. From the earlier empirical results, it can easily be depicted that CSR activities have supportive employees’ perceptions of their organization. OP and organizational commitment positively mediate the relationship between the perceived CSR and creative working environment/behavior. This positive relationship fosters employee pro-environmental behavior which extends positive actions and thoughts of the employees. In other words, participation in CSR activities boosts employee’s engagement toward pro-environmental activities. The mechanism for perceived CSR and employee creative attitude and behavior provides extended micro-level research on OP and organizational commitment, which are very much restricted to direct effect on employee behavior.

The results of serial mediation of employee OP and organizational commitment depicted that they mediate the relationship between the perceived CSR and employee’s creative behavior. The involvement of organizations in CSR activities motivates employees to show commitment, concentration, and enthusiasm at the workplace. Socially responsible activities of the organization develop a sense of achievement and contentment among the employees. CSR activities are the empathic activities of the organization toward its stakeholder, that can be society, environment, or employees, which is an essential driver for OP. The empathetic attitude of the organization is concerned with the wellbeing of the environment and a stakeholder. Organizational CSR activities are the resource that enhances and drives OP, which results in commitment; once they are committed to the organization, they contribute to the role of creative behavior.

### Theoretical Contribution

This study adds the literature on micro-level CSR and workplace creativity in the service industry. First, we investigated the predictors of workplace creativity and analyzed the predictive role of employee perception of CSR activities conducted by the organization. Furthermore, this study highlights CSR as an important predictor of employee creative workplace behavior. Second, this study responds to the research call of previous researchers (Islam et al., 2019; Raza et al., 2021). Furthermore, this article is helpful for advanced research on the psychological mechanism and antecedents of creative work behavior. Furthermore, we attempted to provide additional evidence on how perceived CSR activities positively impact employees’ creative work behavior. This study demonstrates that the CSR activities by the organization increase its employees’ pride and result in organizational commitment. Furthermore, this organizational commitment results in creative work behavior when employees perceive that their organization participates in CSR activities. This study also adds the knowledge of affective emotional theory by explaining how perceived CSR impacts the organization’s pride and employee’s attitude and workplace behavior.

Moreover, this study also contributes to the literature on micro-level CSR, which suggests that employee behavior changes when an organization is involved in CSR activities, which are OP and organizational commitment. Our study is a pioneer in examining the serial mediation of perceived CSR, pride, and organizational commitment, which positively impacts ECB. Through the lens of affective emotional theory, employees reshape their behavior at the workplace, which enhances their pride and commitment, leading to creative behavior.

### Practical/Managerial Implication

This study also provides some managerial implications. The investigation of the study suggests that the CSR activities enhance the employees’ pride and commitment toward organizational goals, which results in employees’ creative behavior. Therefore, an organization should actively step in for the development and enhancement of CSR perception among the employees. For this purpose, organizations should present their CSR activities using different platforms, such as newsletters, social media, such as Facebook, Instagram, and Twitter, and magazines, which are useful in enhancing the perception of CSR activities among stakeholders, especially employees.

Moreover, the organization should develop documentaries and contents related to their CSR activities to improve their perception of environmental and social issues. According to Pérez et al. (2020), storytelling content presents the organization’s efforts to solve social problems and develop a positive image among stakeholders. Furthermore, this study highlights the importance and value of CSR activities, resulting in employee pride and commitment to the organization. If the company incorporates these activities, it would result in the devotion and enthusiasm of employees. Therefore, organizations’ mission and vision statements should reflect their genuine CSR activities. Organizations should conduct training and educational workshops to develop empathy and pro-social behavior among the stakeholders.

## Data Availability Statement

The original contributions presented in the study are included in the article/supplementary material, further inquiries can be directed to the corresponding author/s.

## Author Contributions

SI: conceptualization, methodology, software. LW: supervision, validation, review, and edit. SZ: visualization and investigation. All authors contributed to the article and approved the submitted version.

## Conflict of Interest

The authors declare that the research was conducted in the absence of any commercial or financial relationships that could be construed as a potential conflict of interest.

## Publisher’s Note

All claims expressed in this article are solely those of the authors and do not necessarily represent those of their affiliated organizations, or those of the publisher, the editors and the reviewers. Any product that may be evaluated in this article, or claim that may be made by its manufacturer, is not guaranteed or endorsed by the publisher.
